# RNA-seq of peripheral blood mononuclear cells of congenital generalized lipodystrophy type 2 patients

**DOI:** 10.1038/s41597-021-01040-4

**Published:** 2021-10-13

**Authors:** Yen-Hua Huang, Tzu-Chien Su, Chung-Hsing Wang, Siew-Lee Wong, Yin-Hsiu Chien, Yu-Tai Wang, Wuh-Liang Hwu, Ni-Chung Lee

**Affiliations:** 1 Institute of Biomedical Informatics, National Yang-Ming University, Taipei, Taiwan; 2 Center for Systems and Synthetic Biology, National Yang-Ming University, Taipei, Taiwan; 3 Department of Pediatrics, Children’s Hospital, China Medical University, Taichung, Taiwan; 4 School of Medicine, China Medical University, Taichung, Taiwan; 5 Department of Pediatrics, Ditmanson Medical Foundation Chia-Yi Christian Hospital, Chia-Yi, Taiwan; 6 Department of Pediatrics and Medical Genetics, National Taiwan University Hospital, Taipei, Taiwan; 7 National Center for High-performance Computing, National Applied Research Laboratories, Hsinchu, Taiwan

**Keywords:** Endocrine system and metabolic diseases, Gene expression

## Abstract

Illumina RNA-seq analysis was used to characterize the whole transcriptomes of peripheral blood mononuclear cells (PBMCs) from patients with congenital generalized lipodystrophy. RNA-seq information for seven patients with type 2 congenital generalized lipodystrophy (CGL2; Berardinelli-Seip congenital lipodystrophy, BSCL2) was obtained and compared with similar information for seven age- and sex-matched healthy control subjects. All seven CGL2 patients carried biallelic pathogenic mutations affecting the BSCL2 gene and had clinical symptoms of varying severity. The findings provide the whole-transcriptome signatures of PBMCs of CGL2 patients, allowing further exploration of gene expression patterns/signatures associated with the various clinical symptoms of patients with this disease.

## Abstract (summary)

**Table:** 

Measurement(s)	RNA-Seq • RNA
Technology Type(s)	Illumina HiSeq. 2500 • RNA sequencing
Sample Characteristic - Organism	Homo sapiens

Machine-accessible metadata file describing the reported data: 10.6084/m9.figshare.15022521

## Background & Summary

RNA sequencing (RNA-seq) is a term that describes the use of next-generation sequencing (NGS)-based methodology to target whole transcriptomes. This approach has gained wide popularity in biomedical research, in which profiling of the gene expression levels in samples can be very useful. Compared with other high-throughput transcriptome profiling technologies, such as DNA microarrays, the RNA-seq approach has the advantage of a wider dynamic range of measurement, which enables more sensitive detection of the global gene expression signatures of a given cell population or tissue^1^. Thus, RNA-seq allows for a more precise identification of the gene expression differences of cells when normal and abnormal tissues are compared. Indeed, RNA-seq has become a ubiquitous tool for the study of human diseases, including metabolic disorders, cancers, and infectious diseases^2^. Overall, RNA-seq-identified changes in the transcriptome that are associated with a disease can reveal dysregulation affecting associated biological pathways. The approach also aids in the identification of molecular markers reflecting disease progression.

Congenital generalized lipodystrophy (CGL; CGL1, MIM #608594; CGL2, MIM #269700; CGL3, MIM#612526; CGL4, MIM#613327) is a group of rare autosomal recessive disorders characterized by defects in the biogenesis of lipid droplets; individuals with these genetic diseases lack adipose tissue from early in life due to mutations in *AGPAT2, BSCL2, CAV1, or CAVIN1*^3–8^. Metabolic disorders in CGL patients are the main factors causing morbidity and mortality. Deficiency in adipose mass results in leptin deficiency^9^, leading to the main clinical features of these diseases, including hepatomegaly, muscular hypertrophy, cardiomyopathy, insulin resistance, and hypertriglyceridemia^3^. Furthermore, leptin deficiency might be associated with the lowered immunity frequently observed in CGL patients, as leptin is an important modulator of both the innate and adaptive immune systems. For example, leptin activates polymorphonuclear neutrophils (PMNs), exerts proliferative and antiapoptotic activities on T lymphocytes, affects cytokine production and regulates activation of monocytes and macrophages^10–12^. To date, approximately 500 patients with CGL have been reported in the literature. In Taiwan, type 2 disease (CGL2), which is caused by *BSCL2* mutation, is most common based on surveillance across Asia (73% to 100%)^13–17^.

Compared to CGL1, CGL2 presents with a more severe phenotype, including extensive fat loss, cardiomyopathy, intellectual impairment (50% to 78%), and premature death (15%)^13,18^. Hypertrophic cardiomyopathy (25% of CGL2 patients) often leads to death in the third decade of life. In addition, early onset of liver cirrhosis, renal failure, and recurrent bacterial infection are potential lethal complications. Diagnosis of CGL is based on the patient’s clinical and biochemical phenotype and is usually confirmed by molecular testing^19^. Therapeutic strategies include restriction of total fat intake (20–30% of total dietary energy), treatment with fibric acid derivatives, prescription of n-3 polyunsaturated fatty acids to control hypertriglyceridemia, and standard glucose-lowering treatments^20^. In addition, leptin replacement therapy has been approved since 2014 by the Food and Drug Administration (FDA) for the treatment of severe metabolic abnormalities that result in generalized forms of lipodystrophy^21^.

Clinically, we have observed that some CGL2 patients develop hyperactivity and seizures in late childhood but that others develop metabolic syndrome that is difficult to control with glucose-lowering drugs. To elucidate the processes underlying these different outcomes, the aim in this study was to explore differences in RNA signatures between CGL2 patients and matched control subjects.

## Methods

### Subjects and blood sample preparation

**Fig. 1:**
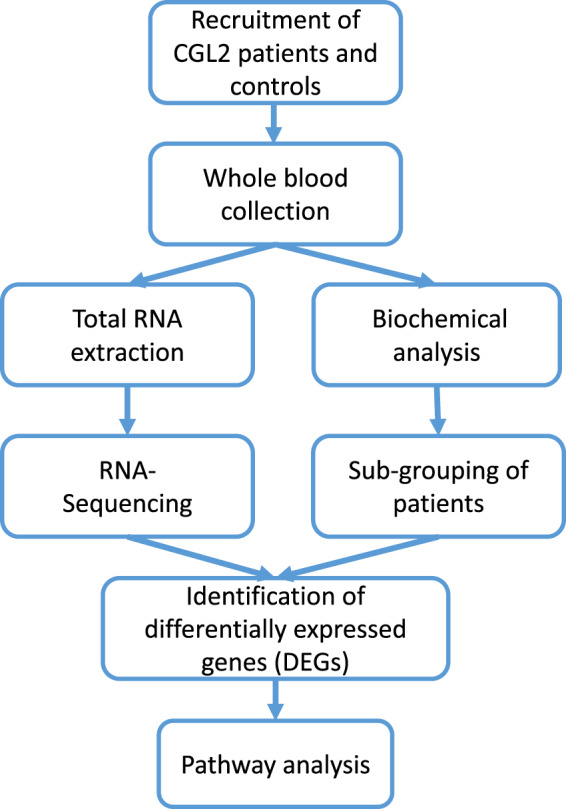
The workflow of this study.

This study investigated differentially expressed genes that might be associated with various subcategories of CGL2 patients and aimed to identify distinct regulatory pathways involved in these various phenotypes. The overall workflow is presented in Fig. 1.

Seven patients (3 female patients and 4 male patients) who had been diagnosed with CGL2 (Table 1) and seven healthy individuals age- and sex-matched with the patients participated in this study; the latter group was used as a control. All enrolled CGL2 patients had been shown to carry *BSCL2* mutation by Sanger sequencing after their initial diagnosis of CGL. Each was further categorized into various subgroups based on age, symptoms, and individual biochemical profiles, namely, child or young adult, normal blood lipid or hyperlipidemia, intellectual disability or not, and diabetes mellitus (DM) or not. Additionally, patients with severe DM or hyperlipidemia, namely, patients 1, 4, 6, and 7, were assigned together into another subgroup, the metabolic syndrome subgroup (presence of insulin resistance or a diagnosis of DM); patients 2, 3, and 5 comprised the “nonmetabolic syndrome” subgroup. Peripheral blood samples were collected after obtaining written informed consent from the participants themselves or one of their parents if the individual was younger than 18 years old. The study protocol was approved by the Institutional Review Board of National Yang-Ming University (IRB number: YM108113E) and the Institutional Review Board of National Taiwan University Hospital (IRB number: 201901028RINB). Informed consent for these data to be openly shared was obtained from all subjects or their guardians, and the participants were warned that RNA-seq data carry some inherent risk of reidentification. To reduce variability, each blood sample was drawn in the morning before the participant broke their fast. Each blood sample was collected into a BD Vacutainer® EDTA tube, and soon after collection, an aliquot from each sample was used for RNA-seq transcriptomic profiling of peripheral blood mononuclear cells (PBMCs).

**Table 1:** Sex, age, and categories of the participants with CGL2 and their BSCL2 mutant alleles.

Patient	Sex	Age	Relation	Relationship	IQ category	Diabetes mellitus	Allele 1	Allele 2
1	F	5 y	N/A	Child/Hyperlipidemia	Normal	No	c.565 G > T (p.G1u189Ter)	c.782dup (p.Ile262HisfsTer12)
2	M	6 y	Sibling	Child/Intellectual disability	Mild	No	c.782dup (p.Ile262HisfsTer12)	c.782dup (p.Ile262HisfsTer12)
3	M	3 y		Child/Intellectual disability	Mild	No	c.782dup (p.Ile262HisfsTer12)	c.782dup (p.Ile262HisfsTer12)
4	F	19 y	Sibling	Young adult/Diabetes Mellitus/Hyperlipidemia/Intellectual disability	Mild	Yes	c.565 G > T (p.G1u189Ter)	c.782dup (p.Ile262HisfsTer12)
5	M	17 y		Young adult/Intellectual disability	Moderate	No	c.565 G > T (p.G1u189Ter)	c.782dup (p.Ile262HisfsTer12)
6	M	16 y	Sibling	Young adult/Diabetes mellitus	Borderline	Yes	c.545_546insCCG (p.Glu182delinsAspArg)	c.565 G > T (p.G1u189Ter)
7	F	13 y		Young adult/Diabetes mellitus/Hyperlipidemia	Borderline	Yes	c.545_546insCCG (p.Glu182delinsAspArg)	c.565 G > T (p.G1u189Ter)

BSCL2 transcript: NM_032667.6

*FIQ was categorized as borderline (71–84), mild (50 to 69), moderate (36–49), severe (20–35), or profound (<20) intellectual disability (mental retardation) according to DSM-IV classification^32^. For patients 6 and 7, who were not available for the IQ test, the severity category was classified as borderline intellectual functioning based on the finding that they underwent vocational high school education with a minimal requirement of support while low normal functioning was observed^33^.

### Preparation of total RNA from PBMCs and RNA-seq analysis

Each whole-blood sample was heparinized, and the samples were used to isolate PBMCs by density gradient centrifugation using LymphoprepTM medium according to the manufacturer’s instructions. Specifically, a sample was diluted with phosphate-buffered saline (PBS, pH 7.4) to double its volume, layered on top of 5 mL of Ficoll-Paque Plus (GE Healthcare, Cat. No. 17-1440-02) and centrifuged for 30 minutes at 400 × g. The PBMC layer was aspirated and centrifuged for collection. The PBMC pellet was treated for 15 minutes with RBC lysis buffer in the absence of light and then centrifuged again followed by washing twice with PBS. Total RNA was extracted using a miRNeasy Kit (Qiagen Cat. No. 74004), followed by DNase I treatment to avoid contamination with genomic DNA. All procedures were according to the manufacturer’s instructions. The fourteen samples containing total RNA from the subject’s PBMCs were submitted to The Genomics Center for Clinical and Biotechnological Applications of National Core Facility for Biopharmaceuticals, which is located at National Yang-Ming University, Taipei. At this facility, RNA quality assessment, RNA integrity assessment, and whole-transcriptome sequencing were carried out. The sequencing library was prepared using a TruSeq Stranded Total RNA Library Ribo-Zero™ Globin Kit (Illumina, San Diego, CA, USA). As the amount of input RNA for the preparation of sequencing libraries was ~1 μg, which is approximately the highest level suggested by the manufacturer’s manual, no positive control, such as SMARTer® Ultra™ Low RNA Kit for Illumina® Sequencing (Takara Bio USA, Mountain View, CA, USA), was used. Paired-end RNA-seq (2 × 100 bases) was carried out using the Illumina HiSeq. 2500 platform.

### RNA-seq data analysis

RNA-seq data analysis was performed to identify differentially expressed genes (DEGs) by assessing the number of reads mapped to the individual gene present in the human reference genome. The workflow of the RNA-seq data analysis was as follows: (1) preprocessing of raw RNA-seq reads and quality validation; and (2) read mapping and normalization.

#### Preprocessing of raw RNA-seq reads and quality validation

**Fig. 2:**
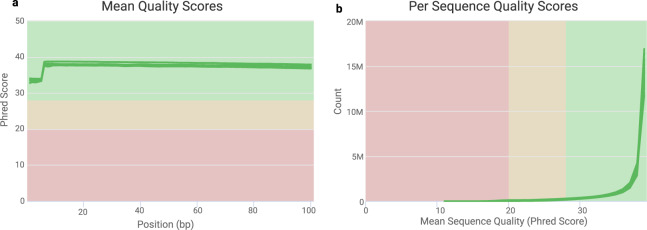
Assessment of the sequence quality scores of the raw FASTQ data. The sequencing quality of the raw FASTQ files was evaluated by using FastQC and then summarized by using MultiQC to create aggregated reports. All 14 FASTQ files were assessed for mean per-base (**a**) and per-sequence (**b**) quality as measured by the Phred score.

During RNA-seq data preprocessing, the quality of the raw RNA-seq reads was evaluated using FastQC^22^ and MultiQC^23^, providing per-base and per-sequence assessments of the sample’s read quality, overall GC contents, and presence of adaptors, overrepresented k-mers and duplicated reads. In addition, Trimmomatic was used to discard low-quality reads, to trim adaptor sequences and to eliminate poor-quality bases^24^. The quality control steps and read trimming were iteratively performed to ensure that all low-quality reads and adaptor sequences were removed as much as possible because they would seriously interfere with the read-mapping step. The quality of the cleaned reads was further assessed by generating per-base box plots using FastQC. Next, MultiQC was applied to create a visualization of the output across the various different samples (Fig. 2), which was used to identify any global trends and/or biases that might affect the sequence quality metrices^23^.

#### Read mapping and normalization

Cleaned reads were mapped to the human reference genome (GRCh38) using the splice-aware alignment tool STAR^25^, followed by BAM file sorting using SAMtools^26^. The RNA-seq read mapping results are summarized in Table 2.

**Table 2:** RNA-seq read statistics. All RNA samples had RIN >7.0 and 260/280 > 1.8.

Sample	NCBI GEO	Paired-end reads (raw)	Paired-end reads (cleaned)	Remaining %	Uniquely mapped reads	Uniquely mapped%	Seq. batch
Control_1	GSM4826885	21,828,683	21,393,525	98.0%	16,810,898	78.6%	1
Control_2	GSM4826886	27,251,284	26,755,597	98.2%	21,118,417	78.9%	1
Control_3	GSM4826887	24,931,372	24,443,108	98.0%	18,260,149	74.7%	1
Control_4	GSM4826888	26,879,004	26,291,723	97.8%	19,457,043	74.0%	1
Control_5	GSM4826889	26,795,914	26,207,057	97.8%	19,711,634	75.2%	1
Control_6	GSM4826890	25,299,512	24,862,820	98.3%	18,692,854	75.2%	1
Control_7	GSM4826891	25,424,700	24,936,311	98.1%	18,407,765	73.8%	1
Patient_1	GSM4826878	24,647,461	24,131,516	97.9%	17,743,976	73.5%	1
Patient_2	GSM4826879	24,757,287	24,332,834	98.3%	18,956,429	77.9%	1
Patient_3	GSM4826880	22,988,027	22,550,204	98.1%	16,698,356	74.0%	1
Patient_4	GSM4826881	25,487,312	25,078,286	98.4%	20,196,651	80.5%	1
Patient_5	GSM4826882	23,639,342	23,108,960	97.8%	17,442,669	75.5%	1
Patient_6	GSM4826883	22,931,749	22,545,880	98.3%	16,741,981	74.3%	1
Patient_7	GSM4826884	24,073,144	23,652,701	98.3%	17,536,718	74.1%	1

**Fig. 3:**
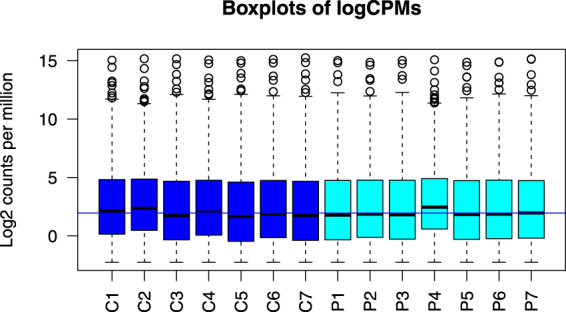
Boxplots of the log2(counts per million) of the cleaned data of all of the RNA-seq.

**Fig. 4:**
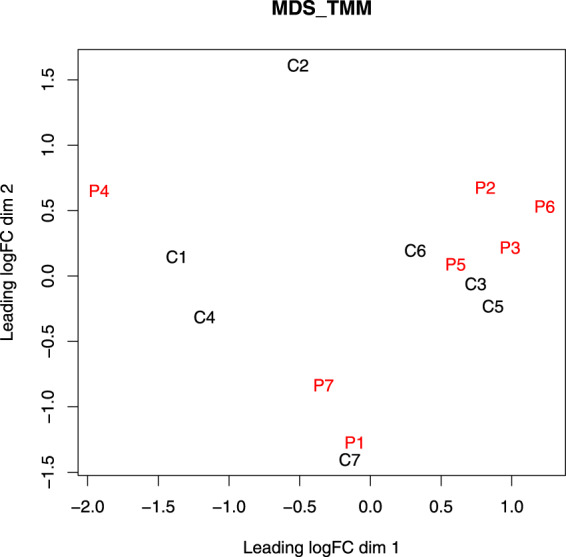
MDS plot for visualizing similarity between the gene expression profiles of different participants.

The read counts mapped to each gene were further normalized by edgeR^27^ using the TMM (trimmed mean of M values) normalization method to calculate various effective library sizes. Boxplots and a multidimensional scaling (MDS) plot were generated to determine whether any unusual expression-pattern similarities between the samples due to batch effects might be present^28^. The boxplots suggested that the variation in the distributions of normalized gene expression levels across different samples was small and that their median values and IQRs (interquartile ranges) were highly similar (Fig. 3). In addition, no batch effect was noted based on the MDS plot, in which the distributions of data points did not reveal any obvious clustering between sibling pairs, between patients, or between normal controls (Fig. 4).

#### Downstream analysis

Statistical tests were carried out using edgeR based on negative binomial differential expression methods and were performed to identify differentially expressed genes (DEGs) when CGL2 patients were compared to age- and sex-matched controls^27^. Based on the instructions provided in the user’s guide of edgeR, we used a paired-sample design to detect DEGs while adjusting for any confounding effects caused by differences in age and sex among the patients.

## Data Records

The FASTQ files for the raw RNA-seq reads in this study have been deposited in NCBI Sequence Read Archive (SRA) under the study accession SRP281919^29^. The raw read count dataset was deposited in the NCBI Gene Expression Omnibus (GEO) under accession number GSE159337^30^ (Table 2). The DEGs of CGL2 patients’ PBMC were deposited in Excel file format in figshare^31^.

## Technical Validation

### RNA integrity assessment

Total RNA was quantified using a Nanodrop ND-1000 spectrophotometer, and its quality was then assessed using an Agilent 2100 Bioanalyzer according to the manufacturer’s instructions. Acceptable quality values are in the range of 1.8−2.2 for A260/A280 ratios and with an RNA integrity number (RIN) of >7.0.

### RNA-seq data quality assessment

The quality of the raw and cleaned RNA-seq reads was evaluated using FastQC^22^, which ensured that the adaptors were removed from the raw reads. This program also verified that the quality of the cleaned RNA-seq reads was suitable for downstream analyses (Fig. 2).

## Data Availability

To finish the RNA-seq data preprocessing, quality validation, and mapping to the human reference genome, only public-domain software but no other custom code was used. These software tools and their versions are listed as follows: 1. FastQC v0.11.8 was used for quality assessment of the raw reads and the trimmed reads of RNA-sequencing data: https://www.bioinformatics.babraham.ac.uk/projects/fastqc/. 2. Trimmomatic 0.38 was used to remove adaptors and do quality trimming: http://www.usadellab.org/cms/?page=trimmomatic. 3. MultiQC 1.0 was used to perform cross-sample quality assessment of the RNA-sequencing reads: https://multiqc.info/. 4. STAR 2.7 was used to map the cleaned RNA-seq reads to the human reference genome assembly, GRCh38: https://github.com/alexdobin/STAR. 5. edgeR 3.30.3 was used to carry out trimmed mean of M values (TMM) normalization for gene expression quantification, and to find the differentially expressed genes (DEGs) between sample groups: https://bioconductor.org/packages/release/bioc/html/edgeR.html.
